# Shared Experiences With Guidewire Application in Chronic Total Occlusion: Technological Advances and Clinical Implications

**DOI:** 10.31083/RCM33469

**Published:** 2025-07-23

**Authors:** Demin Liu, Yaxin Zhi, Wei Sun, Yanan Ma, Shuoyuan Ji, Guoqiang Gu

**Affiliations:** ^1^Department of Cardiology, The Second Hospital of Hebei Medical University, 050000 Shijiazhuang, Hebei, China; ^2^Department of Cardiology, Xingtai People’s Hospital, 054031 Xingtai, Hebei, China

**Keywords:** chronic total occlusion, guidewire technology, interventional treatment, clinical practice, cardiovascular diseases

## Abstract

Chronic total occlusion (CTO) poses a significant challenge in cardiovascular interventional therapy, owing to the complexity and difficulty involved, which limit the effectiveness of traditional treatment methods. Thus, innovative designs and application concepts of guidewires have expanded the possibilities for clinical intervention, resulting in significant improvements in treatment strategies for CTO. Recently, numerous studies have detailed the application experiences of various guidewires in CTO interventional therapy, including the performance characteristics, selection strategies, and differences in clinical outcomes. Nevertheless, despite these advancements, limitations remain in CTO treatment, such as low success rates and the risk of complications. Therefore, this article aims to review the current application experience of CTO guidewires. Moreover, by reviewing existing literature and summarizing clinical practices, this paper explores strategies to optimize guidewire selection and usage to enhance the success rate and safety of CTO interventional therapy and provide practical guidance for clinicians.

## 1. Introduction 

Chronic total occlusion (CTO) refers to lesions in the coronary or peripheral arteries that have been completely occluded for more than three months. Epidemiological studies have shown that approximately 20% of patients undergoing coronary angiography are diagnosed with CTO, which severely impacts their quality of life and increases the incidence of cardiovascular events. The recanalization rate of these lesions is relatively low, posing a challenge for clinical treatment [1, 2]. Despite advancements in CTO percutaneous coronary intervention (CTO-PCI), significant challenges persist, including procedural complexity, suboptimal recanalization rates, and a notable risk of complications. Therefore, enhancing the success rate of CTO therapy remains a critical focus in cardiovascular intervention research. Guidewire technology, a cornerstone of interventional therapy, has evolved in tandem with CTO treatment strategies. Early guidewire technology primarily relies on traditional straight guidewires and balloon dilation techniques. However, with the evolution of interventional techniques, numerous novel guidewires and auxiliary devices have been introduced for CTO treatment. For example, the application of rotational atherectomy guidewires and ultrasound guidance technology has significantly improved the revascularization success rate of CTO [1, 3]. The continuous development of innovative technologies, such as piezoelectric guidewires and AI-assisted navigation systems, not only expands the possibilities for CTO intervention, but also paves the way for future breakthroughs in cardiovascular interventional therapy.

## 2. Guidewire Technology in CTO-PCI: From Characteristic Classification, Strategic Selection to Operational Techniques and Clinical Translation

### 2.1 Classification and Characteristics of Guidewires

#### 2.1.1 Characteristics and Applications of Traditional Guidewires

Traditional guidewires, typically made of stainless steel or polymer materials, are essential in medical interventions due to their moderate flexibility and rigidity, enabling navigation in diverse vascular environments. The design of traditional guidewires allows them to navigate complex vascular anatomies with flexibility while maintaining sufficient pushability for effective lesion traversal. Studies have shown that traditional guidewires are primarily used in fields including cardiovascular intervention, peripheral vascular intervention, and gastrointestinal endoscopy [4, 5, 6]. In cardiovascular intervention therapy, the precise selection of guidewires is a crucial factor for the success of the procedure. However, traditional guidewires face limitations in complex vascular lesions, particularly in cases of severe calcification or tortuosity, due to inadequate support, poor controllability, and a trade-off between stiffness and flexibility. These limitations include inadequate support, poor controllability, and a contradiction between stiffness and flexibility [7]. Therefore, the development of CTO-specific guidewires is of great clinical significance in improving the success rate and safety of surgeries. Among them, the Fielder XT guidewire has been proven to have a high success rate and shorter operation time in CTO interventional procedures. For instance, a retrospective study by Wang *et al*. (2019) [8] involving 1230 CTO patients demonstrated that the Fielder XT guidewire achieved a procedural success rate of 87.8%, compared to 79.0% with conventional guidewires. Additionally, the median procedure time was significantly reduced to 74 minutes when using the Fielder XT guidewire, compared to 83 minutes with traditional guidewires [8].

#### 2.1.2 Innovative Design of Dedicated CTO Guidewires

Specialized CTO guidewires are designed to address the limitations of traditional guidewires in CTO interventions. These guidewires are engineered with higher flexibility and reduced push-through resistance, enabling efficient traversal of complex vascular lesions. Innovative design concepts include the use of composite materials and special tip shapes to enhance the guidewire’s ability to pass through narrow or tortuous vessels. For example, the Fielder XT guidewire, with its core-to-tip tapered tip design, features a conical tip that significantly improves the guidewire’s torque transmission performance and ability to pass through lesions, making it suitable for CTO lesions with microchannel gaps [9]. Similarly, The SUOH 03 guidewire, characterized by its highly flexible tip and low load, possesses excellent flexibility and torque control capabilities, making it suitable for navigating through highly tortuous collateral blood vessels. Recent clinical trial evidence suggests that the Gladius guidewire may offer certain advantages over the Fielder XT guidewire in specific aspects of CTO interventions. The Gladius First Trial compared the Gladius EX guidewire, an intermediate tip-load polymer-jacketed guidewire, with the standard guidewire escalation strategy for antegrade wiring in CTO interventions. The trial demonstrated that the Gladius guidewire could significantly reduce the antegrade wiring time, although the total procedural time, procedural success, safety, and cost were similar between the two groups. This indicates that the Gladius guidewire may provide better time efficiency in certain scenarios of CTO interventions [10]. The continuous development of advanced guidewires, including those with integrated imaging capabilities, is progressively addressing the challenges of CTO intervention, paving the way for more precise and effective treatments [11].

#### 2.1.3 The Impact of Guidewire Materials and Structure on Penetration Force 

The penetration force of a guidewire, a critical performance metric for traversing lesions during interventional procedures, is primarily determined by its materials and structural design, including the core, coating, sheath, and tip configuration [12, 13]. The core, the fundamental component of a guidewire, is typically made of stainless steel or nitinol (nickel-titanium alloy). Stainless steel cores offer excellent pushability and torque transmission, along with strong support, but they lack flexibility and are prone to kinking, making them suitable for penetrating highly resistant lesions such as calcified or fibrotic plaques. In contrast, nitinol cores leverage their superelasticity to navigate tortuous vessels with ease while minimizing the risk of perforation. The sheath design significantly influences the guidewire’s maneuverability, trackability, and visibility. Commonly used sheaths include coil covers, plastic covers, and polymer covers. Coil covers provide enhanced tactile feedback, support, and trackability but increase friction, which may hinder the guidewire’s ability to traverse tortuous, heavily calcified, or occluded lesions. Plastic or polymer covers reduce friction and improve smoothness but may compromise tactile feedback and increase the risk of complications such as perforation or dissection. Modern guidewires often combine multiple sheath designs to optimize performance by balancing their respective advantages and limitations. Guidewire coatings are designed to reduce friction and enhance trackability. Hydrophilic coatings attract water molecules, forming a smooth gel-like surface that minimizes friction and improves trackability. Hydrophobic coatings repel water, creating a wax-like surface that also reduces friction. The tip design is a critical determinant of a guidewire’s controllability and flexibility. Tip engineering integrates three key parameters: tip load, tip taper, and tip shape. High tip load facilitates penetration of resistant or highly stenotic lesions, while low tip load offers a softer, less traumatic tip. Tapered tips provide greater penetration force, and optimal tip shapes enhance steerability. Emerging innovations, such as multi-segment tapered cores and hybrid coatings, further optimize the balance between deliverability and procedural precision. These design elements collectively guide the selection of guidewires tailored to specific lesion characteristics and clinical scenarios (Table 1).

**Table 1.** **Classification and characteristics of market-available and clinical trial-stage CTO guidewires**.

Series/Model	Material	Tip design	Coating	Characteristics	Application	Status
Fielder series (Fielder XT, XT-A, XT-R, Fielder, Fielder FC)	Stainless steel	Cone tip (Fielder XT, XT-A, XT-R, Fielder); non-cone tip (Fielder FC)	Hydrophilic	High torsion transmission, strong microchannel gap ability	Microchannel lesions	On the market
Gaia series (Gaia First/Second/Third)/Gaia Next series	Stainless steel	Micro-cone tip (with preset 45° bend)	Hydrophilic	Excellent control, 1:1 torsion transmission	Angulated lesions, calcified CTO	On the market
Confianza/Conquest	Stainless steel	Cone tip	Hydrophilic	High hardness, strong penetration	Only in straight lesions	On the market
Hornet series (Hornet/Hornet10/Hornet14)	Stainless steel	Cone tip	Hydrophilic	Anti-buckling, precise advancement	Moderate calcified CTO lesions	On the market
Miracle series (Miracle 3/6, Miracle 12)	Stainless steel	Non-cone tip	Hydrophobic	Superior handling performance and enhanced tactile feedback	Lesions at a bend or some tortuosity	On the market
JUDO series (JUDO 1, JUDO 3, JUDO 6)	Stainless steel	Cone tip	Hydrophilic	1:1 control and torsion	Complex severe fibrotic/calcified lesions	On the market
Pilot series (Pilot 50/150/200)	Stainless steel	Non-cone tip	Hydrophilic	Balanced smoothness, tracking performance, and torsional control	Curved, ambiguous CTO lesions, also usable as ADR technique Knuckle wire	On the market
Intravascular lithotripsy (IVL)	Polymer + metal composite	Integrated shock wave emission tip	Hydrophilic	Breaks up calcified plaques through shock waves	Calcified occlusive lesions	In clinical trial
SoundBite^TM^ Crossing System	Polymer sheath	Controllable vibration tip	Hydrophilic	Mechanically vibrates through calcified layers	Complex calcified lesions	In clinical trial
Piezoelectric Wire System	Piezoelectric composite material	Piezoelectric vibration tip	No	Controllable vibration amplitude and frequency	Precise penetration of hard fibrous and calcified lesions	In clinical trial

CTO, chronic total occlusion; ADR, antegrade dissection re-entry.

### 2.2 Strategy for Guidewire Selection

CTO represents one of the most challenging lesion types in interventional therapy, with procedural success heavily reliant on the appropriate selection of guidewires, which are the core instruments for crossing the occlusion and establishing a pathway. Guidewire selection strategies are influenced by factors such as lesion morphology (e.g., calcification severity, vessel tortuosity, and microchannel presence) and patient-specific physiological characteristics (e.g., advanced age, chronic kidney disease, especially dialysis-dependent status, and post-coronary artery bypass grafting (CABG) vascular anomalies).

#### 2.2.1 The Influence of Lesion Pathological Characteristics and Anatomical Morphology on Guidewire Selection

In CTO interventional therapy, the selection of guidewires is critical, which should be combined with the pathological characteristics and anatomical morphology of the lesion [14]. According to the tip load of guidewires, they are divided into three types: low load, medium load and high load. Low-tip load guidewires offer excellent flexibility and maneuverability, making them suitable for non-calcified lesions or tortuous vessels, such as the Fielder XT-R (Asahi Intecc) [15]. Medium-tip load guidewires balance penetration force and torque control, making them suitable for middle-segment lesions with fibrosis, such as Gaia Second (Asahi Intecc) [15]. High-tip load guidewires provide greater penetration force, which are suitable for hard calcified lesions, and the risk of perforation should be guarded when using, such as Conquest Pro 12 (Asahi Intecc) [14, 15]. In addition, guidewires can also be divided into directional guidewires and polymer super-smooth guidewires according to functional design. Directional guidewires feature a shapeable tip and are suitable for bifurcation lesions, such as Sion Black (Asahi Intecc), commonly used in retrograde techniques [15]. Polymer super-smooth guidewires adopt hydrophilic coatings to reduce friction, enabling rapid traversal within the true lumen. In practice, the appropriate guidewire should be selected according to the characteristics of CTO lesions (e.g., fibrous cap morphology, calcification degree, vascular tortuosity) and surgical strategies (e.g., antegrade or retrograde). For example, in antegrade interventions, low-penetration force polymer-coated guidewires may be preferred for lesions without significant calcification; if resistance is encountered, medium- or high-penetration force guidewires may be required. In retrograde interventions, highly flexible guidewires are often chosen to enhance traversal through tortuous collateral vessels.

In terms of specific techniques, the requirements for guidewire characteristics vary: In the antegrade wire escalation (AWE) technique, for short-segment CTO lesions with microchannels, low tip-load guidewires (e.g., Fielder XT-R) are recommended to gently navigate through the microchannels and prevent vascular injury; for calcified lesions, high tip-load guidewires (e.g., Conquest Pro 12) are necessary to enhance penetration, though the risk of perforation must be vigilantly monitored. In the antegrade dissection re-entry (ADR) technique, polymer-coated guidewires (e.g., Pilot 200) are prioritized to maintain the false lumen pathway; when the path is tortuous, “knuckled” guidewires can be employed to form a looped tip to expand the dissection space. In retrograde techniques, for septal collateral vessels, high-torque control guidewires (e.g., Sion Black) are preferred to navigate complex anatomical structures, while for epicardial collateral vessels, the SUOH 03 guidewire is almost exclusively applicable due to its ultra-low tip load and kink resistance, which can reduce the risk of pericardial tamponade. After reaching the distal fibrous cap, the guidewire selection principles for retrograde wire entry (RWE) are the same as those for AWE, and retrograde dissection re-entry (RDR) follows the guidewire selection standards of ADR. These selections are based on the precise matching of guidewire characteristics (tip load, coating material, kink resistance) with technical objectives (penetration needs, flexibility requirements, false lumen control) to achieve the optimal balance between procedural efficiency and safety. In summary, the choice of these specific types of guidewires is determined by the unique challenges posed by each type of CTO lesion, ensuring procedural success and patient safety.

#### 2.2.2 The Impact of Patients’ Physiological Characteristics on Guidewire Selection

Patients’ physiological characteristics are closely associated with lesion complexity, particularly in elderly individuals, those with chronic kidney disease (CKD), and those with a history of CABG. These patient groups often present with vascular calcification, tortuosity, or bypass graft anatomical anomalies, which significantly increase procedural challenges and necessitate the use of guidewires with higher penetration capabilities and aggressive plaque modification strategies. In elderly patients, vascular calcification progresses with age, and concurrent CKD (particularly in dialysis-dependent individuals) exacerbates mineral metabolism disorders, leading to accelerated arterial calcification and the formation of diffuse, rigid plaques [16]. For such lesions, clinicians should prioritize high-tip load guidewires (e.g., Conquest Pro series) or adopt combined use of rotational atherectomy guidewires and shockwave systems (e.g., SoundBite Crossing System) for penetrating densely calcified plaques. Post-CABG patients often develop complex tortuous lesions or anastomotic stenosis due to chronic atherosclerotic processes and surgical trauma in bypass grafts or native vessels [17]. These anatomical anomalies require retrograde intervention techniques paired with high-torque guidewires (e.g., the Gaia series) to improve maneuverability, supplemented by microcatheter support for enhanced guidewire delivery efficiency. Intravascular ultrasound (IVUS) plays a pivotal role in guiding interventions for such complex cases. IVUS enables precise assessment of calcification distribution, true lumen trajectory, and plaque burden, thereby informing guidewire path selection and plaque modification strategies (e.g., directional atherectomy or focused shockwave energy delivery). This approach reduces perforation risks and improves true lumen recanalization rates. In summary, individualized guidewire selection must integrate patient age, comorbidities, and vascular morphological features. The synergistic application of high-penetration guidewires, plaque modification technologies, and imaging guidance is essential to optimize the safety and efficacy of CTO interventions.

### 2.3 Operating Techniques in Guidewire Technology

In CTO interventions, guidewire manipulation requires three key skills: steering, drilling, and penetration. These skills are critical for improving procedural success rates and ensuring patient safety. Steering techniques demand precise control of the guidewire to navigate along the intended path, requiring exceptional hand-eye coordination and a deep understanding of vascular anatomy. Drilling techniques focus on utilizing the guidewire’s penetration force to get through complex lesions or narrow segments, while penetration techniques are primarily used to accurately access the true lumen in specific cases, such as CTO lesions. Proficiency in the performance characteristics of different guidewire types, combined with the use of auxiliary tools such as microcatheters, balloons, and intravascular imaging technologies, can significantly enhance the success rate of guidewire traversal through lesions. Studies have shown that rigorous simulation training and the accumulation of practical experience can effectively improve physicians’ guidewire manipulation skills [18].

#### 2.3.1 Guidance and Manipulation Techniques of Guidewires

Considering the characteristics of the lesion, the judicious selection of a guidewire is the primary and crucial step in interventional surgery. For example, in moderate-difficulty CTO lesions, the use of a polymer-coated, medium-stiffness tipped guidewire has been shown to reduce the number of guidewire passages, shorten the procedural duration, and decrease the volume of contrast agent required, thereby enhancing the efficacy and safety of the intervention [19]. In a comprehensive clinical trial involving a cohort of 260 patients, a significant correlation was observed between the use of a tapered-head guidewire and an increased rate of procedural success [20]. Additionally, data analysis revealed no significant correlation between the diameter and surface coating of the guidewire tip and the efficacy in traversing CTO lesions [20]. When using percutaneous or hydrophilic-coated ultra-slippery guidewires, it is imperative to remain vigilant to prevent inadvertent penetration of the vascular architecture or injury to branch termini. Following guidewire advancement, true lumen positioning should be confirmed through donor artery angiography (e.g., contralateral injection or ipsilateral retrograde injection via a microcatheter), with IVUS optionally employed to enhance guidance precision. Subintimal balloon dilatation is subsequently utilized to create a controlled dissection plane, enabling targeted re-entry into the true lumen under imaging guidance. When employing the ADR technique, it is essential to select stents that match the vessel diameter and control the expansion pressure. Optimal procedural guidance should prioritize perfusion angiography via contralateral injection or retrograde ipsilateral microcatheter injection, complemented by the Stingray balloon system or dual/triple-lumen microcatheters (e.g., Recross) to achieve targeted re-entry. If guidewire passage remains challenging after ADR, the following techniques can be considered: Spontaneous True Lumen Re-entry with Guidewire (STAR), which involves the “knuckling” maneuver to naturally re-enter the true lumen at a side branch with the guidewire tip; Assisted False Lumen Re-entry with Balloon (AFR), which uses balloon dilation to create an artificial intimal window within the dissection to facilitate guidewire exchange. Additionally, local injection of collagenase to degrade fibrous plaque components can also promote guidewire exchange [21].

Concurrently, the efficacy of guidewire utilization is often enhanced through its integration with adjunctive instruments, thereby enhancing the therapeutic success rate. In the context of complex percutaneous interventions, a synergistic approach involving the guidewire, support catheters, microcatheters, and other ancillary devices has been demonstrated to significantly improve the efficacy and safety of crossing vascular occlusions [22]. For instance, the combination of the Stingray balloon system with dual-lumen or triple-lumen microcatheters can achieve precise re-entry guided by retrograde angiography, or navigate to the distal end of the CTO lesion along the retrograde guidewire trajectory. However, despite its advantages, the limited steerability of the guidewire and the propensity for deformation at the wire tip can result in increased procedural time and radiation exposure. To address these challenges, Colangelo* et al*. [23] successfully employed the ‘Balloon-Assisted Tip-In’ technique, which has demonstrated its effectiveness in addressing these issues. Furthermore, the application of a semi-compliant balloon *ex vivo*, positioned adjacent to the microcatheter, serves to stabilize the microcatheter without impeding blood flow at its distal tip. This technique facilitates the introduction of the guidewire into the anterograde microcatheter. Additionally, in interventional cardiology, the integration of guidewire technology with ultrasound guidance systems has been proven to enhance precision in guidewire navigation and positioning. Consequently, this approach not only reduces the technical challenges associated with the procedure but also minimizes the risk of procedural complications [24]. In the realm of interventional cardiology, technological advancements have led to the development of innovative guidewire designs, such as adjustable guidewire systems, which are now being implemented in clinical practice. These systems are designed to be modulated based on real-time feedback, thereby optimizing the efficacy of guidewire utilization [25]. Therefore, the judicious combination of guidewires with other instruments not only enhances the efficacy of treatment but also decreases the risks to patients.

#### 2.3.2 Typical Operational Steps and Precautions

In the execution of guidewire manipulations, adherence to established protocols and precautions is paramount for ensuring procedural safety and efficacy. Prior to the intervention, it is imperative for physicians to select the appropriate guidewire and catheter based on the individual patient’s clinical profile and the specific surgical objectives. During guidewire insertion, maintaining the correct angle and force is essential to minimize the risk of vascular or tissue injury. Throughout the procedure, it is imperative for the operator to vigilantly monitor the advancement of the guidewire to prevent entrapment or fracture. As the guidewire navigates through stenotic regions, real-time imaging modalities such as IVUS or fluoroscopy are essential to ensure precise positioning and facilitate unimpeded traversal. Postoperatively, the integrity of the guidewire and catheter should be inspected to confirm the absence of residual foreign bodies and prevent complications (Table 2).

**Table 2.** **Standard operating procedures for guidewire manipulation**.

Step	Key actions	Tools/Techniques	Precautions
1. Lesion assessment	Evaluate cap morphology	Imaging catheter	Avoid excessive contrast use
2. Guidewire insertion	Select tip shape/stiffness per lesion type	Microcatheter support	Monitor torque response to prevent kinking
3. Traversal	Gentle drilling/steering motions	Rotational atherectomy (if calcified)	Stop if resistance exceeds 3 g force
4. True lumen confirmation	Dual angiography or IVUS validation	Orthogonal fluoroscopic views	Avoid subintimal expansion without imaging
5. Complication management	Entrapment: Rotate gently; Fracture: Retrieve fragment	Snare/wire winding	Immediate imaging for foreign body localization

IVUS, Intravascular ultrasound.

Due to the complexities of CTO lesions, challenges such as guidewire entrapment, fracture, or misdirection are more likely during manipulation. These complications can lead to procedural failure or patient morbidity. In cases of guidewire entrapment, gentle rotation of the guidewire or the use of a more delicate guidewire for adjunctive guidance can help navigate through the stenosis. Prudent manipulation of the guidewire, specifically by avoiding excessive rotation, is instrumental in preventing guidewire entrapment. In the event of guidewire fracture, it is imperative to halt the procedure immediately and initiate imaging studies to pinpoint the site of the break. Subsequently, appropriate protocols must be employed to address the foreign body retrieval effectively [26]. The fracture of a guidewire can be addressed using techniques such as wire winding, basket retrieval, or stent extrusion. Stent extrusion is particularly useful when the guidewire fractures within the vessel and stent implantation is required to maintain vessel patency. Methods to prevent guidewire fracture include timely exchange of the guidewire and withdrawal of the supporting guidewire before releasing the stent when using guidewire support. In cases of misdirection, it is crucial for physicians to remain calm, quickly assess the position of the guidewire, and adjust based on imaging results. Additionally, regular participation in training and simulation exercises can help doctors improve their ability to respond to emergencies and ensure effective management in complex clinical situations. By conducting a thorough analysis of intraoperative challenges and devising targeted strategies, the safety and efficacy of guidewire technology can be markedly enhanced.

#### 2.3.3 Algorithm for Entry Technique Selection in CTO Interventions

The selection of CTO-PCI techniques is based on two key factors: the clarity of the proximal fibrous cap and the length of the lesion, with the goal of achieving safe and efficient procedures through a stepwise strategy while reducing radiation exposure and contrast media usage. Specifically, if the proximal fibrous cap is clearly visible and the lesion is short (e.g., <20 mm), antegrade wiring is the preferred technique due to its direct approach and high success rate; for longer or ambiguous lesions, polymer-sheathed guidewires (such as the Fielder XT) are necessary, although they may enter the subintimal space, necessitating re-entry or parallel wire techniques to correct the path; if the proximal cap is unclear and cannot be confirmed by imaging (e.g., IVUS), retrograde techniques are required, with RWE for short lesions and RDR for long lesions to improve success rates; when both antegrade and retrograde approaches fail, hybrid techniques (such as controlled antegrade and retrograde subintimal tracking-CART) can be employed. Algorithms like the FE Global CTO Algorithm provide a structured decision-making framework to optimize technique selection, prioritizing less invasive and time-efficient strategies to reduce the risk of complications and enhance procedural efficiency.

### 2.4 Recent Research Findings and Technological Advances

#### 2.4.1 Clinical Trial Results of Novel Guidewire Technologies

With the advent of biotechnology, a plethora of specialized guidewires for CTO have been introduced into clinical practice, thereby enhancing the procedural success rates. Research indicates that polymer-coated guidewires possess not only reduced surface friction but also augmented penetrating capabilities and torque control, enabling them to navigate through the proximal cap of fibrocalcific lesions and the narrow, tortuous channels of the lesion with ease. An analysis of 7575 CTO patients who underwent antegrade PCI in 47 centers by Michaella Alexandrou *et al*. [27] demonstrated that the exclusive use of polymer-sheathed guidewires could improve procedural success rates from 85.7% to 94.3% and diminish the risk of perforation from 3.2% to 2.2%. When calcification is present in CTO lesions, conventional guidewires often encounter difficulty in antegrade traversal. A novel shockwave guidewire system, the SoundBite Crossing System, offers a new approach for antegrade revascularization of CTO lesions. The SoundBite Crossing System employs a source console to generate shockwaves that propagate forward through the guidewire, continuously impacting the calcified lesion, ultimately enabling penetration through the CTO lesion and access to the distal true lumen [28]. The research conducted by Eric Therasse* et al*. [29] has validated the effectiveness and safety of the SoundBite Crossing System in facilitating the recanalization of infringingly artery CTO lesions. The PROSPECTOR study (n = 52) demonstrated that the SoundBite Crossing System achieved a technical success rate of 92.3% in calcified CTO lesions, with a perforation rate of 9.6% and no major adverse cardiovascular events (MACE) occurring within 30 days [29]. Currently, clinical studies pertaining to the application of the SoundBite Crossing System in coronary CTO recanalization are ongoing, and it is anticipated that this system will offer a novel treatment option for patients with coronary CTO in the future. Furthermore, the intravascular piezoelectric guidewire system has recently garnered significant attention. This system incorporates innovatively the principle of the piezoelectric effect into guidewire technology, transmitting mechanical vibrations to the guidewire tip to facilitate the disruption and recanalization of CTO lesions. The device offers controllable vibration amplitude and frequency, enabling operators to easily penetrate the tough fibrous cap and calcified lesions within CTO without compromising the integrity of surrounding normal vascular tissues. It serves as an adjunct to other interventional products in navigating through the lesion site, thus enhancing the procedural efficiency of PCI treatment for patients with CTO lesions. Currently, this guidewire system is in the clinical trial phase, and its potential for widespread clinical application in addressing the formidable challenge of CTO lesions is highly anticipated.

#### 2.4.2 Prospects of Guidewire-Imaging Integration

The combination of guidewire technology and imaging is becoming a crucial trend in the realm of interventional medicine. With advancements in imaging technology, particularly the incorporation of three-dimensional visualization and deep learning algorithms, the navigation of guidewires within complex anatomical structures has become more precise. Notably, recent research has developed an automated catheter segmentation approach based on deep learning, which achieves high-precision detection of catheters and guidewires in cerebral angiography [30]. Athanasios Rempakos and his team [31] have developed and validated a machine learning model that can predict the likelihood of successful recanalization of CTO lesions using a primary antegrade wire escalation strategy. The application of this technology not only enhances the safety of interventional procedures but also holds the potential to advance the development of robot-assisted interventional technologies, enabling physicians to better monitor guidewire dynamics during surgery. In the future, with continuous advancements in imaging technology, guidewire technology will be integrated with various image-guided techniques, leading to more intelligent interventional treatment plans and ultimately improving patient outcomes and safety.

#### 2.4.3 Ongoing Prospective Studies and Randomized Controlled Trials

In addition to the completed studies, several ongoing prospective studies and randomized controlled trials (RCTs) are currently underway in the field of CTO intervention, aiming to further validate the clinical efficacy and safety of various guidewire techniques and interventional strategies. The ISCHEMIA-CTO Study is assessing whether CTO-PCI improves quality of life (QoL) and reduces MACE at 6 months, providing insights into the potential benefits of CTO recanalization in asymptomatic patients and those with angina symptoms. The NOBLE-CTO Study (NCT03392415) compares the effects of CTO-PCI and optimal medical therapy (OMT) on all-cause mortality and quality of life in patients with CTO lesions. The ORBITA-CTO Study (NCT05142215) enrolls patients who have undergone 3 months of optimal medical therapy, comparing the effects of CTO-PCI versus a sham procedure on angina relief and investigating the impact of CTO recanalization on patients with vascular complications. The CTO-HF Trial (NCT05632653) assesses whether CTO-PCI improves survival rates and reduces heart failure-related hospitalizations compared to OMT. Lastly, the CTO-ARRHYTMIA Study (NCT04542460) evaluates the impact of PCI versus OMT on the incidence of arrhythmias in patients with CTO lesions. Collectively, these ongoing studies are expected to provide additional evidence to guide clinical practice and further optimize treatment strategies for CTO interventions.

#### 2.4.4 Future Directions in Guidewire Technology Development 

Technological advancements have led to a diverse range of guidewire types and designs, providing clinicians with more options to address various patient conditions. The future trajectory of guidewire technology is predominantly aimed at enhancing flexibility, safety, and intelligent functionalities. The application of innovative materials, such as MXenes, is propelling the performance of guidewires to new heights. These materials are characterized by their exceptional biocompatibility and mechanical properties, rendering them ideally suited for addressing the demands of increasingly complex clinical scenarios [32]. Furthermore, the development of smart guidewires is accelerating, with the integration of sensors and artificial intelligence technology enabling real-time monitoring of their status within the body and providing feedback to assist doctors in optimizing their operational strategies. The emergence of these smart guidewires signifies a shift towards more personalized and precise guidewire technology. In summary, with the continuous emergence of new technologies, the future of guidewire technology will become more diversified, capable of meeting the needs of various clinical scenarios and providing better treatment outcomes for patients.

## 3. Conclusions 

This review comprehensively examines current CTO guidewire technologies, covering their classification, characteristics, selection strategies, and operational techniques. We have analyzed recent research advancements and identified future development directions. By synthesizing clinical practices and literature, we emphasize that optimized guidewire selection and usage can significantly enhance the success rate and safety of CTO interventional therapy, offering practical guidance for clinicians and promoting technological progress in CTO treatment.

Despite significant progress in treating CTO, further optimization and innovation remain essential. Future efforts should focus on multicenter, prospective studies to validate the effectiveness and safety of various guidewire techniques in clinical practice. This would help clarify the optimal use cases for different types of guidewires across diverse lesion types and patient profiles, thereby facilitating the development of personalized treatment strategies. Additionally, physician training and skill enhancement are crucial, with the establishment of standardized training systems aiding in increasing the adoption and proficiency of guidewire techniques. Moreover, interdisciplinary collaboration should be encouraged, integrating advancements in imaging, interventional techniques, and basic medical sciences to collectively propel advancements in the field of CTO treatment. In the future, multicenter studies should aim to quantify the success rates, procedural times, and complication profiles of different guidewire techniques. By incorporating patient-specific factors such as calcification severity and vessel tortuosity, evidence-based strategies for guidewire selection can be established, ultimately improving clinical outcomes.
